# MicroRNAs as Diagnostic Biomarkers in Primary Central Nervous System Lymphoma: A Systematic Review and Meta-Analysis

**DOI:** 10.3389/fonc.2021.743542

**Published:** 2021-09-17

**Authors:** Xiaohong Zheng, Parker Li, Qianqian Dong, Yihong Duan, Shoubo Yang, Zehao Cai, Feng Chen, Wenbin Li

**Affiliations:** ^1^Department of Neuro-Oncology, Cancer Center, Beijing Tiantan Hospital, Capital Medical University, Beijing, China; ^2^Clinical Medicine, Shanghai Jiao Tong University School of Medicine, Shanghai, China; ^3^Department of General Medicine, Ningbo Medical Center, Li Huili Hospital, Zhejiang, China; ^4^Guanghua School of Stomatology, Sun Yat-sen University, Guangzhou, China

**Keywords:** primary central nervous system lymphoma, microRNAs, meta-analysis, diagnosis, brain tumor

## Abstract

**Background:**

Diagnosing primary central nervous system lymphoma (PCNSL) remains a challenge. MicroRNAs (miRNAs) are promising noninvasive markers for the identification of PCNSL. The present study aims to assess the diagnostic value of miRNAs for PCNSL patients as biomarkers.

**Methods:**

We systematically searched PubMed, Embase, and the Cochrane library from inception to January 31, 2021. The pooled sensitivity, specificity, positive likelihood ratio (PLR), negative likelihood ratio (NLR), diagnostic odds ratio (DOR), together with the summary receiver operator characteristic (SROC) curve, and the area under the SROC curve (AUC) value were used to estimate the overall diagnostic performance. We used Q statistic and I^2^ to test heterogeneity and used subgroup analyses to investigate the source of heterogeneity. The statistical analyses were independently performed by two investigators using Stata 14.0 and Revman 5.3.

**Results:**

In total, 11 studies from 6 records were included in the current meta-analysis with 281 PCNSL patients and 367 controls. Our statistical analysis demonstrated that the pooled sensitivity, specificity, PLR, NLR, DOR, and AUC were 0.91 (95% CI 0.84–0.95), 0.88 (95% CI 0.84–0.91), 7.48 (95% CI 5.71–9.78), 0.11 (95% CI 0.06–0.19), 70 (95% CI 35–142), and 0.90 (95% CI 0.87–0.92), respectively. The studies had substantial heterogeneity (I^2^ = 54%, 95% CI 0–100). Two subgroup analyses were conducted based on the type of specimen and miRNAs profiled.

**Conclusions:**

This meta-analysis indicated that miRNAs were suitable as noninvasive diagnostic biomarkers for PCNSL with high accuracy. In addition, both cerebrospinal fluid-based and blood-based miRNAs assays for PCNSL detection were considered reliable for clinical application. MicroRNA-21 assays also seemed to be more accurate in the diagnosis of PCNSL. Good quality studies with large samples should be conducted to verify our results.

## Introduction

Primary central nervous system lymphoma (PCNSL) is a rare but aggressive form of extranodal non-Hodgkin lymphoma (NHL) solely affecting the central nervous system, including the brain, spinal cord, leptomeninges, and eyes (1). With an incidence of 0.44 per 100,000, PCNSL accounts for 1 to 2% of all NHLs and 2 to 7% of all primary central nervous system (CNS) tumors (2). PCNSL has a dismal prognosis, indicated by its low survival rates of 29.9% in 5 years and 22.2% in 10 years (3). Given its highly aggressive nature and poor prognosis, early diagnosis is essential for the successful treatment and improved prognosis of PCNSL.

However, current diagnosis methods are still wanting. Neuroimaging magnetic resonance imaging (MRI) is the most common method, but it cannot give definitive diagnosis due to its inability to differentiate PCNSL from other CNS diseases (such as gliomas, demyelinating entities, vasculitis, neurosarcoidosis, and infections) (4). The histological examination of tumor specimens, preferably obtained through stereotactic needle biopsy, is the standard diagnostic procedure for PCNSL (5). However, it can fail due to unreachable lesion location or inadequate specimen quality for diagnosis, and it entails the risk of hemorrhage and neurologic damage. Less invasive liquid biopsy plays an important role in the diagnosis of PCNSL. When PCNSL is suspected on radiological grounds, a lumbar puncture is often performed for cerebrospinal fluid (CSF) investigation. The analysis of CSF includes cytomorphology, flow cytometry, and immunoglobulin gene rearrangement, but these methods have low diagnostic yield (6–8). Currently, microRNAs (miRNAs) obtainability from blood or CSF have shown promising prospect as markers for the liquid biopsy analysis of PCNSL, not only for improving diagnostic yield but also for monitoring therapy response (9, 10).

MiRNAs are an identified class of noncoding single-strand RNA molecules that inhibit gene expression at post-transcriptional level by binding to the 3’ untranslated regions of mRNA transcripts to interfere with translational initiation or trigger the degradation of mRNAs (11, 12). MiRNAs expression is deregulated in various malignancies including leukemia and lymphoma (11). Evidence has shown that miRNAs expression profiling becomes increasingly important for CNS cancer as a useful diagnostic tool (13–15).

Many researchers have investigated the diagnostic value of miRNAs in PCNSL detection but have obtained inconsistent results (9, 10, 16, 17). Therefore, we are currently unsure about the diagnostic accuracy of these miRNAs assays, which will substantially hinder the establishment of their clinical application in PCNSL diagnosis. In this systematic review and meta-analysis, we aim to address this problem by investigating and summarizing the results in the 11 studies on miRNAs as biomarkers in PCNSL diagnosis to find a common conclusion and test their reliability.

## Methods

### Search Strategy

This systematic review and meta-analysis are reported in accordance with the Preferred Reporting Items for Systematic Reviews and Meta-Analyses (PRISMA).

We selected relevant studies published up to January 31, 2021, by searching Embase, PubMed, and Cochrane Library. We used the following key terms: “Primary central nervous system lymphoma” and “MicroRNA.” The complete search used for PubMed was as follows: (Primary central nervous system lymphoma OR PCNSL OR Primary CNS lymphoma OR Primary central lymphoma) AND (pre miRNA[Title/Abstract] OR pre-miRNA[Title/Abstract] OR Small Temporal RNA[Title/Abstract] OR stRNA[Title/Abstract] OR Temporal RNA, Small[Title/Abstract] OR RNA, Small Temporal[Title/Abstract] OR pri miRNA[Title/Abstract] OR pri-miRNA[Title/Abstract] OR miRNA, Primary[Title/Abstract] OR Primary miRNA[Title/Abstract] OR MicroRNA, Primary[Title/Abstract] OR Primary MicroRNA[Title/Abstract] OR miRNA[Title/Abstract] OR RNA, Micro[Title/Abstract] OR Micro RNA[Title/Abstract] OR miRNAs[Title/Abstract] OR MicroRNA[Title/Abstract] OR (“MicroRNAs”[Mesh])). In addition, we searched the reference lists of the identified articles and previous meta-analysis to identify other potential studies.

### Inclusion and Exclusion Criteria

Diagnostic miRNA studies were considered eligible if they met the following criteria: 1) focused on patients with PCNSL, including HIV-negative and HIV-infected; 2) measured the miRNA expression in tumor tissue, cerebrospinal fluid, or blood fluids; 3) reported data relating to the diagnostic prediction and predictive performance, including parameters such as specificity, sensitivity, and area under the receiver operating characteristic (ROC) curve; and 4) related to the diagnostic value of miRNAs for PCNSL diagnosis.

Studies were excluded if they were 1) reviews, letters, reports, conference abstracts or papers, mail articles, and editorials; 2) lacking of essential data for the pooled calculation; or 3) duplicate publications.

### Data Extraction

Data extraction was independently performed by two authors (XZ and PL) using a standard protocol. Discrepancies were resolved by consensus and consultation with a third investigator. We extracted the following data from each selected study: first author, year, country of publication, sample size, age, type of specimen, assay method, cutoff value, miRNA profiled, sensitivity, specificity, and areas under the curve (AUCs).

### Statistical Analysis

Statistical analysis was independently performed by two investigators (XZ and PL). Disagreements were resolved by a third investigator (FC). The diagnostic performance of miRNAs was evaluated by calculating aggregate sensitivity, specificity, positive likelihood ratio (PLR), negative likelihood ratio (NLR), diagnostic odds ratio (DOR), together with the summary receiver operator characteristic (SROC) curve, and the area under the SROC curve (AUC) value (18). In order to ensure high diagnostic informativeness, a positive test result requires an LR that is greater than 10, and a negative test result requires an LR that is less than 0.1. A moderate informational value can be achieved with PLR values of 5–10 and NLR values of 0.1–0.2. PLR values of 2–5 and NLR values of 0.2–0.5 have a very small informational value. The value of a DOR ranges from 0 to infinity. The higher the value of the DOR, the better its discriminatory test performance. The following guidelines have been suggested for the interpretation of intermediate AUC values: low (0.5 >= AUC < 0.7), moderate (0.7 > =AUC < 0.9), or high (0.9 >= AUC <= 1) diagnostic accuracy.

Heterogeneity among the studies was evaluated through the chi-square test (Q statistic) and I^2^ statistic. A P value lower than 0.10 for the Q test or I^2^ greater than 0.50 indicated significant heterogeneity. Subgroup analyses based on the type of specimen and miRNAs profile were used to identify sources of heterogeneity. We assessed the methodological quality of included studies with the revised Quality Assessment of Diagnostic Accuracy Studies (QUADAS-2) (19). The QUADAS-2 tool was specifically developed to assess the applicability and risk of bias of studies; it contains a seven-item checklist with three options for each item: yes, unclear, no. A study will be classified as having concerns if the risk of bias or the applicability concerns were assessed as high, or if both were assessed as unclear. To test for publication bias, we constructed effective sample size funnel plots *versus* the log diagnostic odds ratio and did Deeks’ regression test of asymmetry (20), with a P value lower than 0.10 showing statistical significance.

We used the MIDAS module for STATA 14.0 (StataCorp, College station, TX, USA) for the diagnostic accuracy analysis (sensitivity, specificity, PLR, NLR, DOR, AUC) and subgroup analysis. We used Revman 5.3 to do the Quality Assessment of Diagnostic Accuracy Studies.

## Results

### Characteristics of the Studies

**Figure 1:**
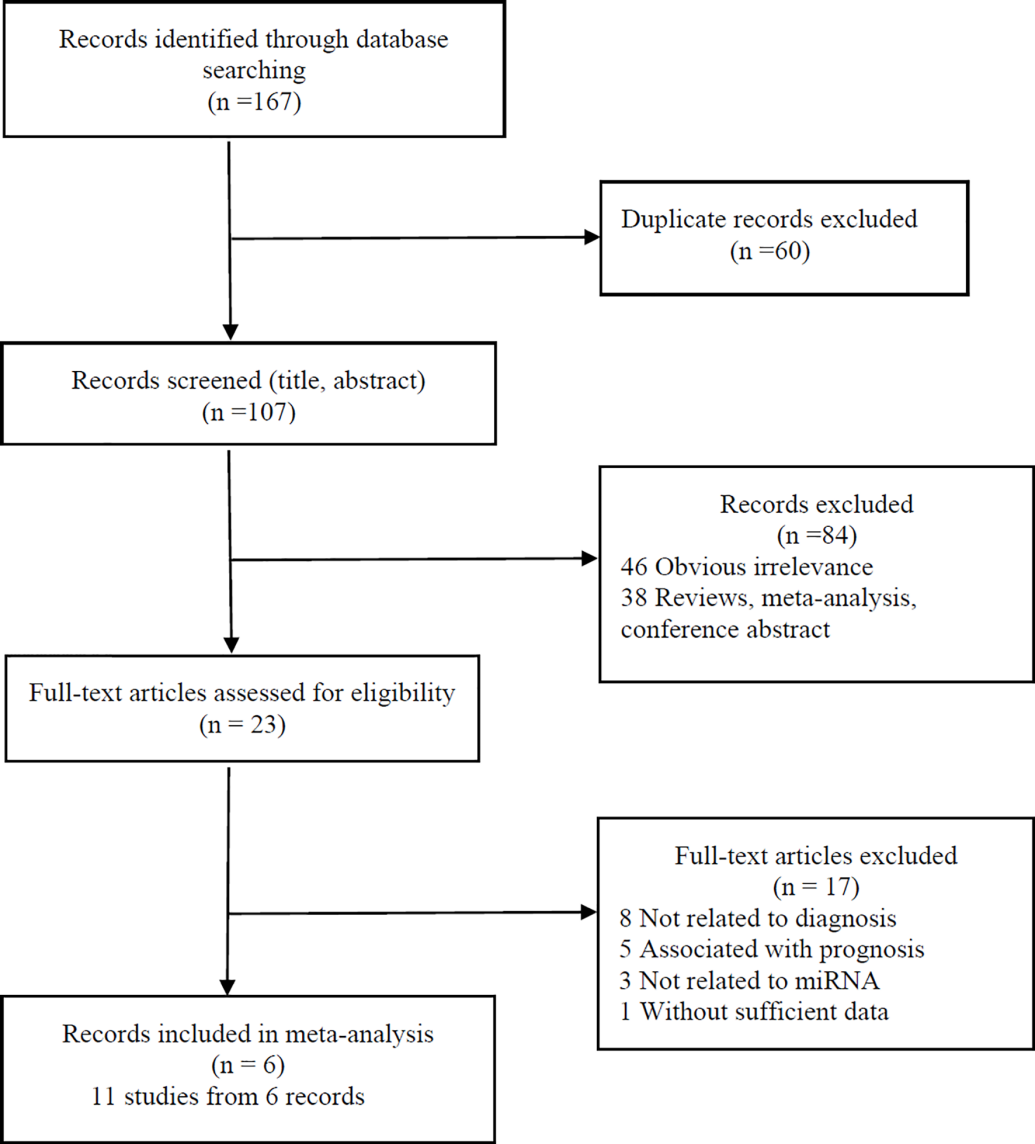
Flow diagram of study selection process.

We initially identified 167 records. After eliminating the duplicate articles and applying the inclusion and exclusion criteria, six of them (with data for 281 PCNSL patients and 367 controls) (9, 10, 16, 17, 21, 22) were included in our analysis (**Figure 1**). The classification and features of the included studies are shown in **Table 1**. The six included records, published between 2011 and 2019, were conducted in different countries: two in Germany, two in China, one in the USA, and one in Poland. One record (21) recruited HIV-infected patients; the remaining records were HIV-negative patients. In total, these records contained 11 studies focusing on miRNAs for PCNSL detection. Six of them measured the miRNAs expression level in CSF, four in blood, and one in brain tumor. Levels of miRNAs expression were detected by quantitative real-time polymerase chain reaction (qRT-PCR). For diagnostic performance, multiple miRNAs assays were investigated in 3 of the 11 studies, while single miRNA assays were investigated in the remaining 8.

**Table 1:** Characteristics of enrolled studies.

First author	Year	Contrary	Sample size (case *vs* control)	Age (range)	Type of specimen	Assay method	Cut-off	miRNA profiled	Sensitivity %	Specificity %	AUC
Baraniskin (9)	2011	Germany	23 *vs* 30	PCNSL: mean 64(42-77)	CSF	qRT-PCR	8	miR-21	95.7	83.3	0.94 (95% CI 0.886-1.00)
				Control: mean 51.2(24-80)							
				PCNSL: mean 64(42-77)	CSF	qRT-PCR	1.4	miR-19b	95.7	83.7	0.98 (95% CI 0.955-1.01)
				Control: mean 51.2(24-80)							
				PCNSL: mean 64(42-77)	CSF	qRT-PCR	2.5	miR-92a	95.7	80	0.97 (95% CI 0.925-1.01)
				Control: mean 51.2(24-80)							
				PCNSL: mean 64(42-77)	CSF	qRT-PCR	\	miR-19b, miR-21, and miR-92a	95.7	96.7	\
				Control: mean 51.2(24-80)							
Mao (10)	2014	China	56 *vs* 122	\	Serum	qRT-PCR	Mean expression level	miR-21	86	90	0.930 (95% CI 0.881-0.979)
			37 *vs* 88	\	Serum	qRT-PCR	Mean expression level	miR-21	84	90	0.916 (95% CI 0.852-0.979)
Thapa (21)	2014	USA	20 *vs* 45	PCNSL: median 40.2	Serum	qRT-PCR	0.109	miR-222	80	82	0.792 (95% CI 0.663-0.920)
				Control: median 36.4							
Baraniskin (22)	2018	Germany	55 *vs* 11	PCNSL: mean 65.1(41-87)	CSF	qRT-PCR	Mean expression level	miR-30c	90.9	85.5	0.859 (95% CI 0.703-1.016)
				Control: mean 60.6(20-79)							
Yang (16)	2019	China	25 *vs* 25	PCNSL: median 56.6(36-69)	plasma	qRT-PCR	Mean expression level	miR-21	96.3	91.7	0.971 (95%CI:0.933-1.000)
				Control: median 57.2(43-68)							
Zajdel (17)	2019	Poland	35 *vs* 23	PCNSL: median 62(31-82)	brain tumor	qRT-PCR	Median expression level	miR-let-7b and miR-155	96	91.8	0.988
				Control: median 47(18-78)							
			30 *vs* 23	PCNSL: median 62(31-82)	CSF	qRT-PCR	Median expression level	miR-19b, miR-21,and miR-92a	63.3	80.8	\
				Control: median 47(18-78)							

Vs, versus; PCNSL, primary central nervous system lymphoma; CSF, cerebrospinal fluid; qRT-PCR, quantitative real-time polymerase chain reaction; AUC, area under the curve; 95% CI, 95% confidence interval; \, not available.

### Diagnostic Accuracy

**Figure 2:**
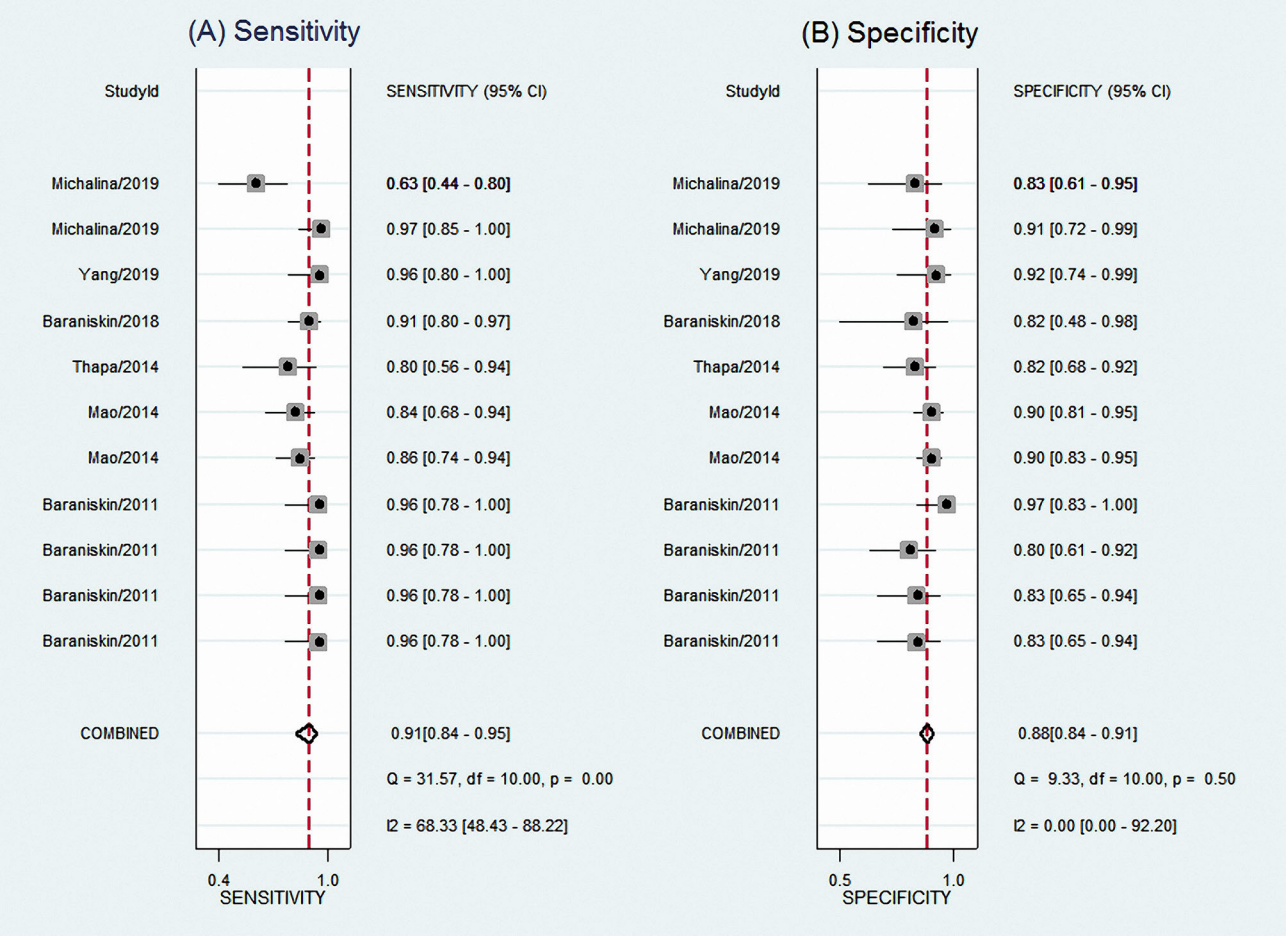
Forest plots of sensitivities **(A)** and specificities **(B)** for total miRNA levels of 11 studies in the diagnosis of PCNSL. PCNSL, primary central nervous system lymphoma.

**Figure 3:**
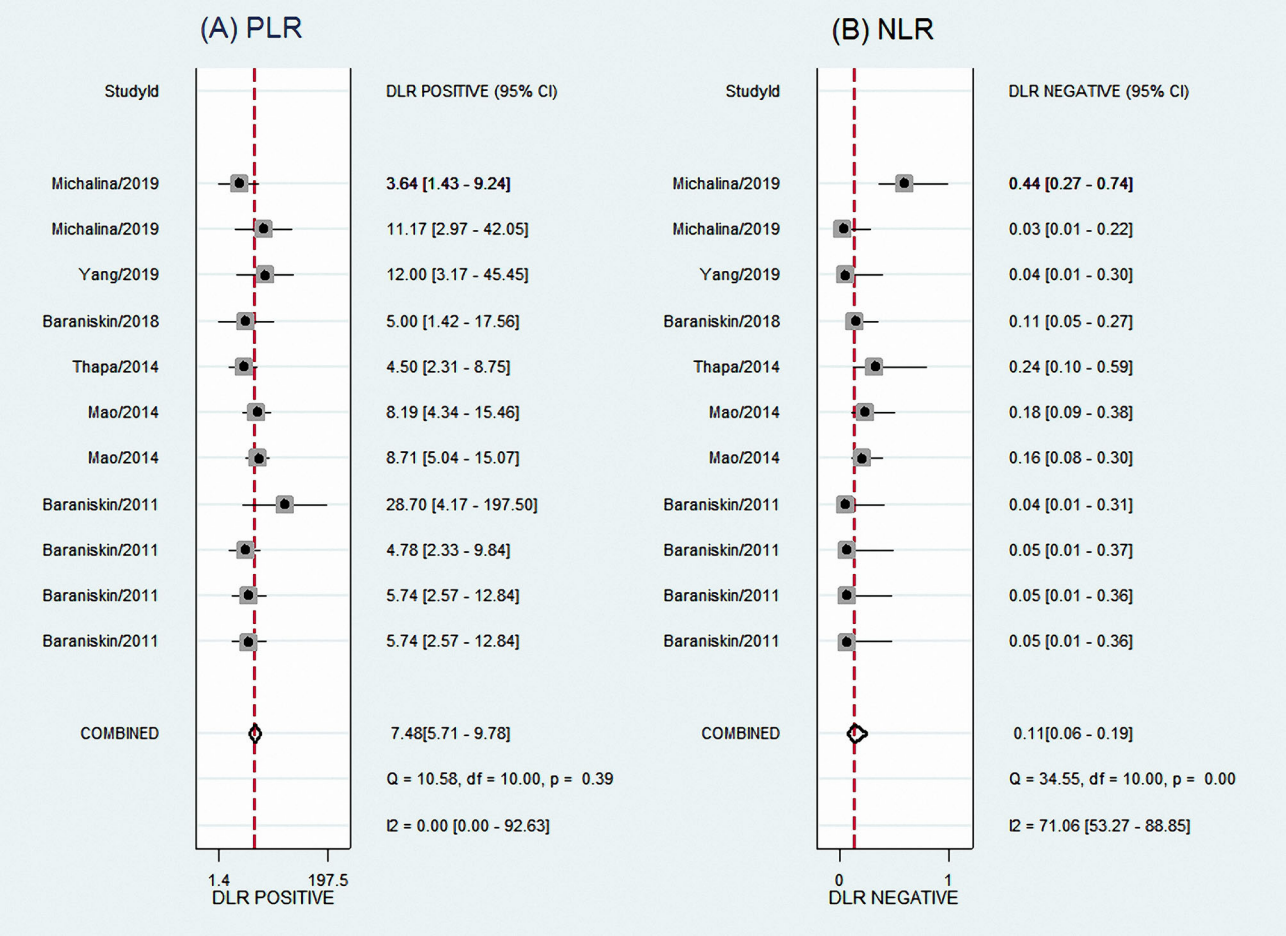
Forest plots of PLR **(A)** and NLR **(B)** for total miRNA levels of 11 studies in the diagnosis of PCNSL. PLR, positive likelihood ratio; NLR, negative likelihood ratio; PCNSL, primary central nervous system lymphoma.

**Figure 4:**
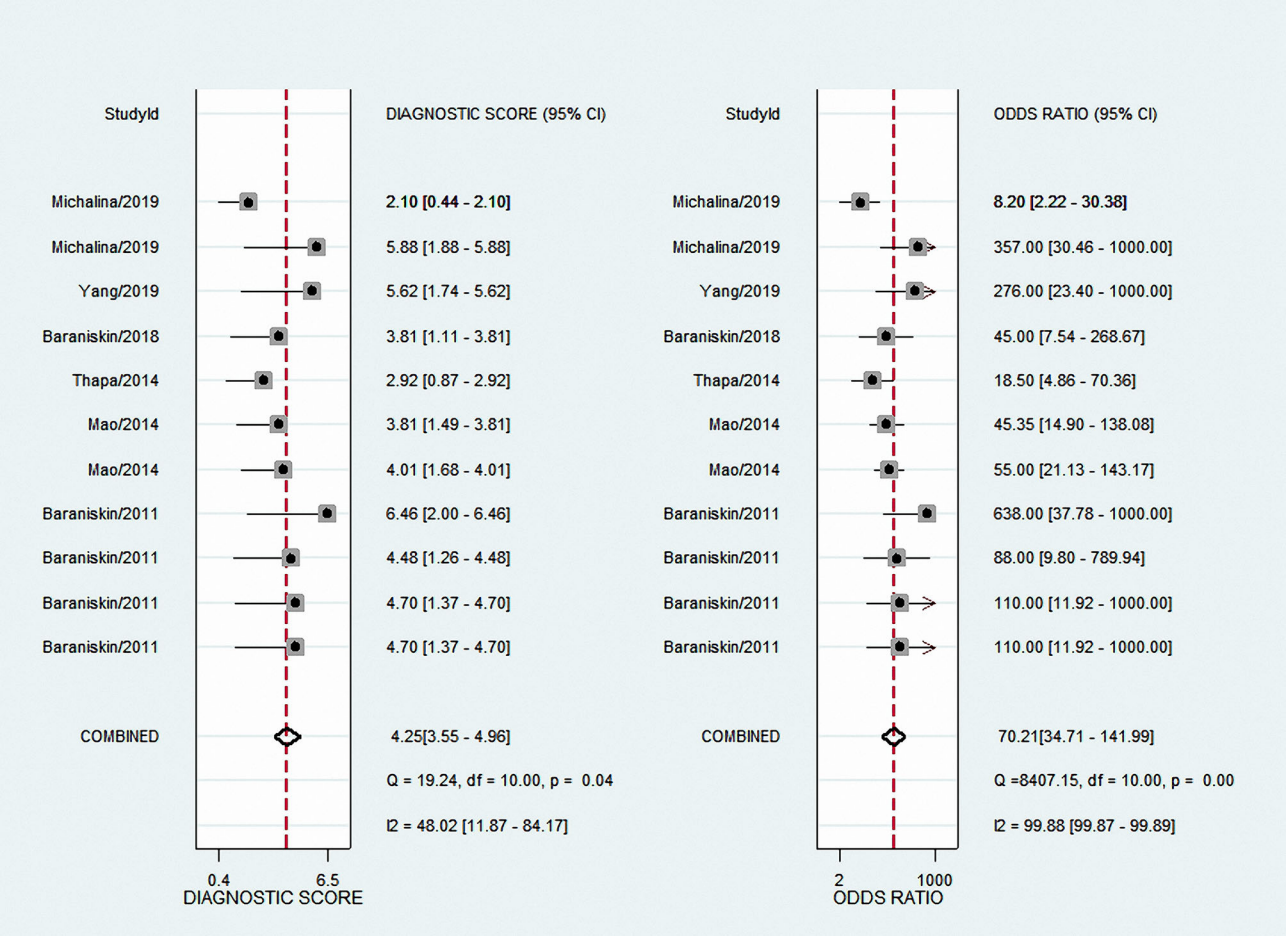
Forest plots of DOR for total miRNA levels of 11 studies in the diagnosis of PCNSL. DOR, diagnostic odds ratio; PCNSL, primary central nervous system lymphoma.

**Figure 5:**
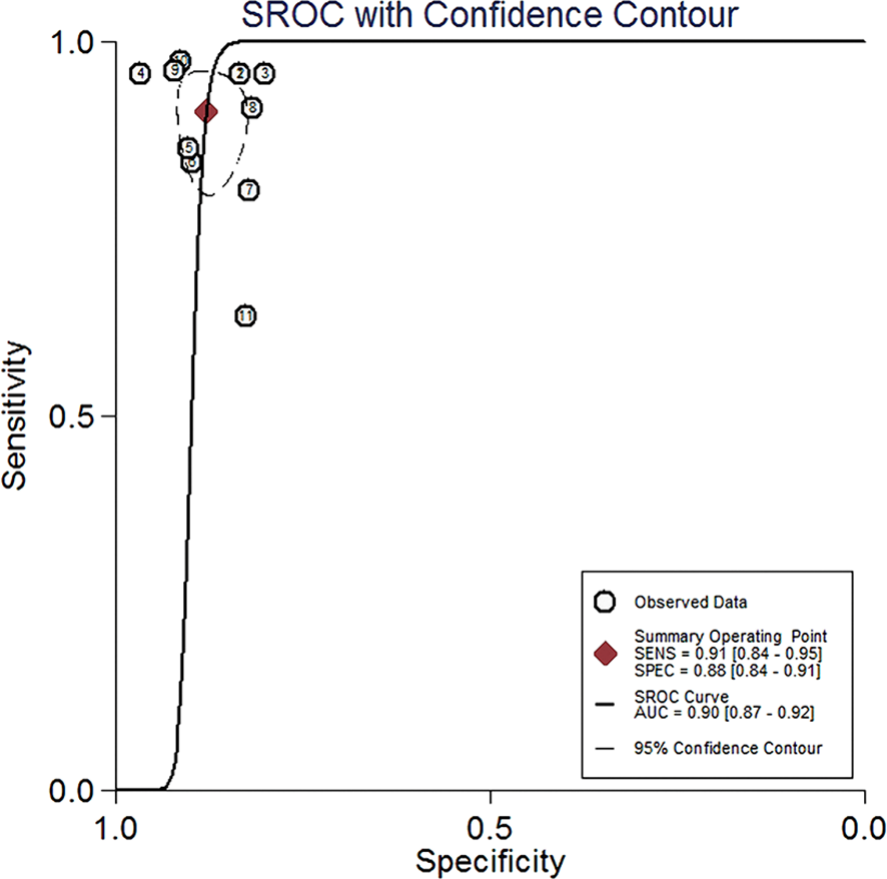
Summary ROC curve with confidence around mean operating sensitivity and specificity point. ROC, receiver operator characteristic.

The results showed an aggregate sensitivity of 0.91 (95% CI 0.84–0.95) and a pooled specificity of 0.88 (95% CI 0.84–0.91), as shown in **Figure 2**. The NLR was 0.11 (95% CI 0.06–0.19), and the PLR was 7.48 (95% CI 5.71–9.78) (**Figure 3**), suggesting a moderate diagnostic informational value. The pooled DOR was 70 (95% CI 35–142) (**Figure 4**), indicating good discriminatory test performance. The summary ROC plot showed that the diagnostic aggregate value of the AUC was 0.90 (95% CI 0.87–0.92) (**Figure 5**), suggesting high diagnostic accuracy and a promising future for miRNAs as biomarkers for PCNSL detection. Substantial heterogeneity existed among the studies (overall I^2^ for the bivariate model was 54%, 95% CI 0–100). A meta-analysis of the diagnostic odds ratio showed the presence of high heterogeneity with a Q value of 8407.15 (*p* < 0.001) and I^2^ of 99.88%. Similarly, the heterogeneity for sensitivity was high with a Q value of 31.57 (*p* < 0.001) and an I^2^ of 68.33%. Therefore, a random-effects model was applied to each meta-analysis.

### Subgroup Analysis

Subgroup analyses were conducted to identify sources of heterogeneity. We performed a subgroup analysis based on the type of specimen, but we only investigated blood and CSF because there was only 1 study about brain tumor tissue specimen in the 11 studies. As shown in **Table 2**, the performance of miRNAs in blood for the diagnosis of PCNSL showed a pooled sensitivity of 0.86 (95% CI 0.79–0.91) and a pooled specificity of 0.89 (95% CI 0.85–0.92). The combined PLR and NLR were 7.79 (95% CI 5.55–10.93) and 0.15 (95% CI 0.10–0.24), respectively, which indicated a moderate diagnostic informational value. Its combined DOR was 50 (95% CI 27–93) and AUC was 0.94 (95% CI 0.91–0.96), suggesting relatively high diagnostic accuracy. The performance of miRNAs in CSF for PCNSL detection showed an aggregate sensitivity of 0.92 (95% CI 0.81–0.97) and an aggregate specificity of 0.85 (95% CI 0.78–0.90). Its pooled PLR was 6.10 (95% CI 4.10–9.20) and NLR was 0.09 (95% CI 0.04–0.24), revealing a moderate positive and high negative diagnostic informativeness value. The pooled DOR was 65 (95% CI 21–207) and the AUC was 0.88 (95% CI 0.85–0.91), displaying moderate diagnostic accuracy. Thus, both CSF-based and blood-based assays could be considered reliable for clinical application.

**Table 2:** Diagnostic performance of miRNAs in patients with PCNSL.

Analysis	Overall	CSF-based	Blood-based	MiR-21	Non-miR-21
No. of studies	11	6	4	4	7
SEN (95% CI)	0.91 (0.84-0.95)	0.92 (0.81-0.97)	0.86 (0.79-0.91)	0.89 (0.82-0.93)	0.91(0.81-0.96)
I^2^ (*P* value)	68.33 (<0.001)	81.10 (<0.001)	0.00 (0.41)	21.35 (0.28)	79.17 (<0.001)
SPE (95% CI)	0.88 (0.84-0.91)	0.85 (0.78-0.90)	0.89 (0.85-0.92)	0.89 (0.85-0.93)	0.86 (0.79-0.90)
I^2^ (*P* value)	0.00 (0.50)	0.00 (0.50)	0.00 (0.47)	0.00 (0.70)	0.00 (0.43)
PLR (95% CI)	7.48 (5.71-9.78)	6.10 (4.10-9.20)	7.79 (5.55-10.93)	8.39 (5.88-11.97)	6.38 (4.25-9.58)
I^2^ (*P* value)	0.00 (0.39)	0.00 (0.48)	0.00 (0.36)	0.00 (0.76)	0.00 (0.30)
NLR (95% CI)	0.11 (0.06-0.19)	0.09 (0.04-0.24)	0.15 (0.10-0.24)	0.13 (0.08-0.20)	0.10 (0.04-0.24)
I^2^ (*P* value)	71.06 (<0.001)	82.79 (<0.001)	0.00 (0.42)	10.51 (0.34)	81.25 (<0.001)
DOR (95% CI)	70 (35-142)	65 (21-207)	50 (27-93)	66 (34-127)	62 (21-189)
I^2^ (*P* value)	99.88 (<0.001)	99.80 (<0.001)	82.26 (<0.001)	60.29 (0.06)	99.85 (<0.001)
AUC	0.90 (0.87-0.92)	0.88 (0.85-0.91)	0.94 (0.91-0.96)	0.95 (0.93-0.97)	0.91 (0.88-0.93)

No., number; SEN, sensitivity; SPE, specificity; PLR, positive likelihood ratio; NLR, negative likelihood ratio; DOR, diagnostic odds ratio; AUC, area under the curve; 95% CI, 95% confidence interval; CSF, cerebrospinal fluid.

We also conducted a subgroup analysis based on the miRNAs profile, including miR-21 and non-miR-21 assays (**Table 2**). The pooled sensitivity, specificity, PLR, and NLR of non-miR-21 assays were 0.91 (95% CI 0.81–0.96), 0.86 (95% CI 0.79–0.90), 6.38 (95% CI 4.25–9.58), and 0.10 (95% CI 0.04–0.24), respectively, with a DOR of 62 (95% CI 21–189) and an AUC value of 0.95 (95% CI 0.88–0.93). The diagnostic performance of miR-21 was better for PCNSL detection. The pooled sensitivity, specificity, PLR, and NLR of miR-21 assay were 0.89 (95% CI 0.82–0.93), 0.89 (95% CI 0.85–0.93), 8.39 (95% CI 5.88–11.97), and 0.13 (95% CI 0.08–0.20), respectively, with a DOR of 66 (95% CI 34–127) and an AUC value of 0.95 (95% CI 0.93–0.97). Therefore, both miR-21 and non-miR-21 can be used as a diagnostic biomarker for PCNSL, but miR-21 might have a higher diagnostic value.

The results of the subgroup analyses suggested that the source of heterogeneity may be associated with the type of specimen and miRNAs profiled.

### Quality Assessment

**Figure 6:**
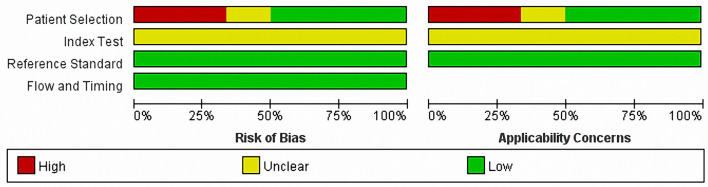
QUADAS-2 risk of bias and applicability concerns graph showing review authors’ judgments about each domain as percentages of included studies. QUADAS-2, the revised Quality Assessment of Diagnostic Accuracy Studies.

**Figure 7:**
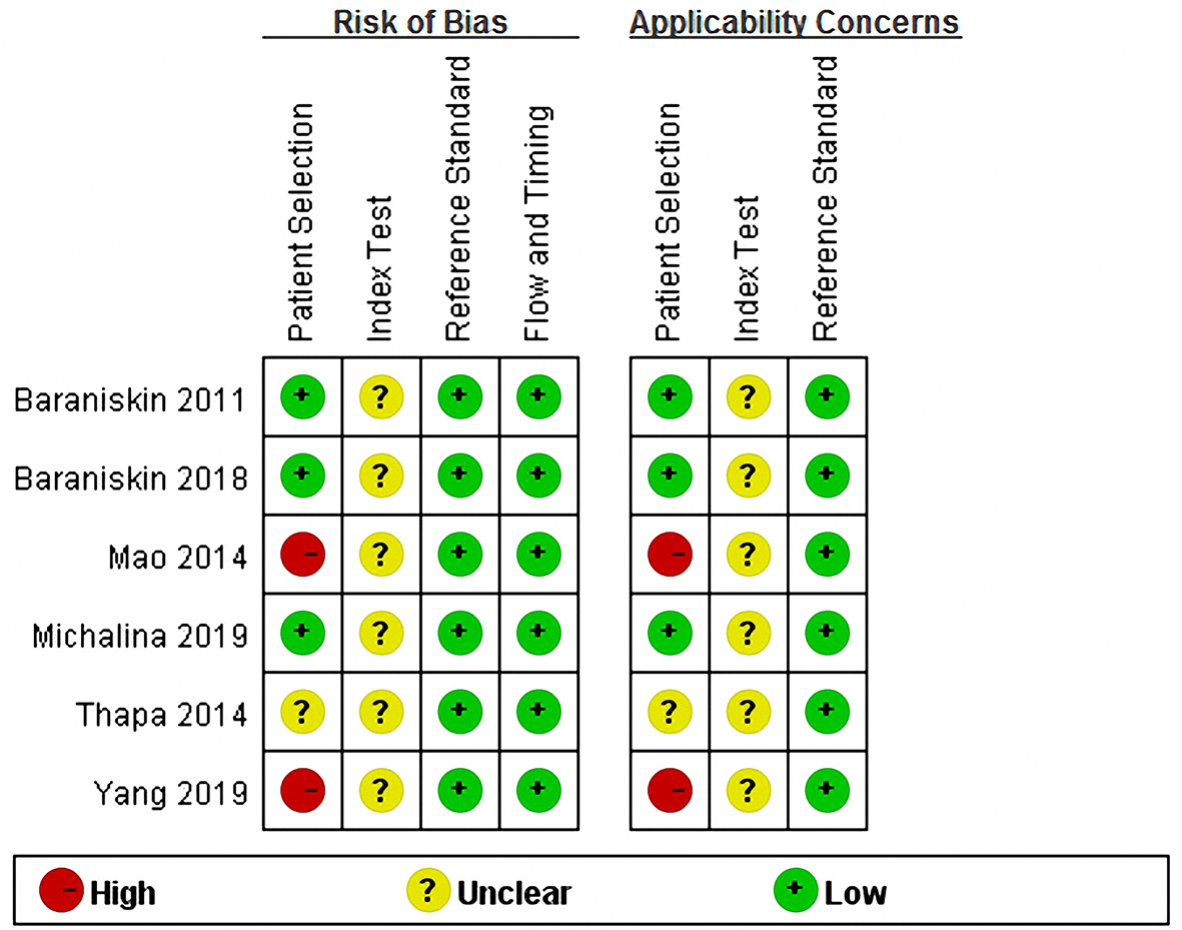
QUADAS-2 summary of risk of bias and applicability concerns showing review authors’ judgments about each domain for each included study. QUADAS-2, the revised Quality Assessment of Diagnostic Accuracy Studies.

The methodological quality of the included studies was evaluated using the QUADAS-2 framework. **Figures 6** and **7** summarized the overall risk of bias and applicability concerns. For patient selection, the risk of bias and applicability concerns were generally low as most studies included consecutive series of patients with suspected or confirmed PCNSL. However, for the index test, both risk of bias and applicability concerns were unclear primarily because in most studies, it was unclear whether the index test was performed blinded to the results of the reference tests. Besides, contributing to the risk of bias alone, we were also unsure whether the thresholds used had been prespecified, with some using study-derived thresholds. For the reference standard, the risk of bias and applicability concerns were both assessed as low because histopathological examination was appropriately used as the reference standard in all the studies. The flow and timing were rated as low risk of bias. In total, all the included records displayed moderate to relatively high quality according to the QUADAS-2 criteria.

### Publication Bias

**Figure 8:**
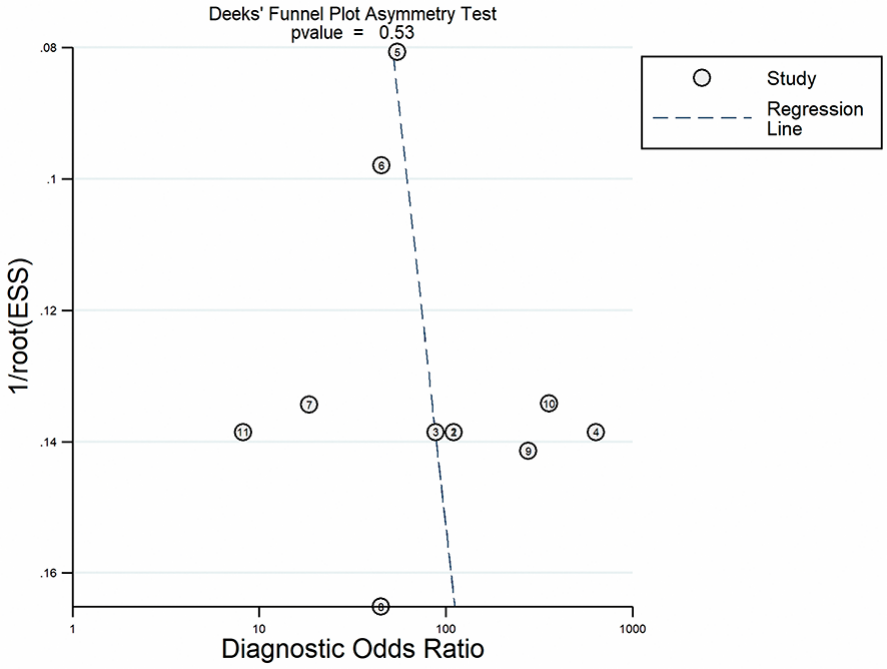
Linear regression test of funnel plot asymmetry.

The Deeks’ funnel plot asymmetry test was conducted to explore publication bias in the current study (**Figure 8**). The p value of 0.53 indicated that no bias existed in the publication.

## Discussion

PCNSL is an aggressive tumor with a life expectancy of 3–5 months without treatment. Unfortunately, the definite diagnosis of PCNSL is often delayed by averagely 3 months after the initial symptoms appear for non-acquired immunodeficiency syndrome patients (23) because diagnosing PCNSL remains a challenge. Clinical and radiological features may suggest the suspected diagnosis of PCNSL, but they are not definitely diagnostic. Diagnosis of PCNSL is usually established by stereotactic brain biopsy. However, this invasive procedure has a complication rate of 8.5%, including hematomas, seizures, or brain edema (24). Although the cytology or flow cytometry of CSF or vitreous fluid is less invasive than stereotactic brain biopsy (25), these are only positive in case of leptomeningeal or ocular involvement, respectively. In search of a novel diagnostic approach with high accuracy and limited risks to shorten the delay of PCNSL diagnosis, many researchers have turned their eyes to miRNAs, which can stably present in several body fluids including CSF and blood and may serve as non-invasive biomarkers for PCNSL diagnosis (13, 14).

However, the researchers have found inconsistent results. Baraniskin et al. (9) reported the analysis of miR-21 (95.7% sensitivity and 83.3% specificity), miR-19b (95.7% sensitivity and 83.7% specificity), and miR-92a (95.7% sensitivity and 80.0% specificity) in CSF, either alone or combined (95.7% sensitivity and 96.7% specificity), identified PCNSL from controls with high accuracy. Nonetheless, Zajdel et al. (17) performed a combined assessment of the three miRNAs in discriminating PCNSL from nonmalignant brain lesions and found a specificity of 80.8% and a sensitivity of 63.3%, indicating lower diagnostic accuracy than what Baraniskin et al. presented. Mao et al. (10) examined miR-21 in the blood and showed a sensitivity of 86.0% and a specificity of 90.0% in the detection of PCNSL. Yang et al. (16) found higher diagnostic accuracy of miR-21 in blood, with a specificity of 91.7% and a sensitivity of 96.3%. Therefore, we found that it is necessary to assess the potential applicability and reliability of miRNAs as diagnostic biomarkers for PCNSL patients.

To the best of our knowledge, this is the first study to specifically evaluate the value of miRNAs as diagnostic biomarkers in PCNSL. Although one meta-analysis study about miRNAs as diagnostic biomarkers in CNS cancers has been published (26), it focused on all types of CNS cancers, including but not limited to PCNSL. In addition, few studies of PCNSL were included in that meta-analysis study. It could not support further evaluation on the relationship between miRNAs and PCNSL consequently, which needs more PCNSL cases to confirm the final findings. Another relevant systematic review (27) did not perform a formal meta-analysis on the diagnostic accuracy of the markers for CNS lymphoma in blood and CSF, and focused on various markers at the same time including CXCL13, interleukins-6, -8, and -10, soluble CD19, and so on, not just miRNAs. All in all, the specific evaluation on miRNAs as diagnostic biomarkers in PCNSL has not been explored to our knowledge.

Overall, our results indicated that miRNAs are suitable as diagnostic biomarkers for PCNSL with high accuracy, with 0.91 for sensitivity, 0.88 for specificity, 70 for DOR, and 0.90 for AUC. The result of the subgroup analysis on the type of specimen indicated that the performance of miRNAs in CSF (sensitivity of 0.92 and DOR of 65) was better than that in blood (sensitivity of 0.86 and DOR of 50) for PCNSL detection. However, the combined specificity and AUC of miRNAs in blood for the diagnosis of PCNSL were higher than CSF-based miRNAs assays, where the specificity increased from 0.85 to 0.89, and the AUC increased from 0.88 to 0.94. Thus, both CSF-based and blood-based assays could be considered reliable for clinical application with relatively high diagnostic accuracy. A review (13) described that miRNAs in blood and CSF had important diagnostic advantage because they were contained in protective vesicles derived from cell membrane and resistant to RNase digestion, exhibiting a remarkable stability. In line with our conclusion, the review showed that blood and CSF as relatively noninvasive specimens have been widely used in PCNSL detection.

We conducted another subgroup analysis based on the miRNAs profiled (miR-21 and non-miR-21). The performance of miR-21assays for PCNSL detection showed an aggregate sensitivity of 0.89, specificity of 0.89, DOR of 66, and an AUC value of 0.95, which were better than non-miR-21 assays (sensitivity of 0.91, specificity of 0.86, DOR of 62, and AUC of 0.91). In agreement with our results, many articles have confirmed the high diagnostic value of miR-21 in both blood and CSF for PCNSL. For example, Baraniskin et al. (9) were the first to find CSF miR-21 a highly accurate diagnostic marker for PCNSL (AUC 0.94). Another study (10) indicated a high diagnostic value of serum miR-21 for PCNSL (AUC 0.93). Significant positive correlation of miR-21 was found between serum and CSF in this study (Pearson correlation: r^2^ = -0.396, p = 0.001). A recent study (28) demonstrated that miR-21 combined with small nuclear RNA fragments of RNU2-1f in CSF had high diagnostic accuracy, resulting in AUC of 0.987 with a sensitivity of 91.7% and a specificity of 95.7%.

Our study can provide reference to the clinicians regarding the reliability of miRNAs assays at diagnosing PCNSL. Likelihood ratios and post-test probabilities provide information about the likelihood that a patient with a positive or negative test actually has PCNSL or not. In our study, both the overall likelihood ratio and post-test probability were moderate (**Figure S1**). A positive likelihood ratio of 7 indicates that a person with disease is seven times more likely to have a positive test result than a healthy person is. Given a pretest probability of 20%, the post-test probability for a positive test result is 65%. Likewise, a negative likelihood ratio of 0.11 reduces the post-test probability to 3% for a negative test result.

Two included records (9, 17) showed the diagnostic accuracy of combined miRNAs panels. Baraniskin et al. (9) reported that combined miR-21, miR-19b, and miR-92a analyses yielded a higher discriminatory diagnostic value than any single one. However, the diagnostic accuracy of the combined three miRNAs Zajdel et al. (17) presented was lower than what Baraniskin et al. reported. The reason for this discrepancy most probably lies in the reference groups. The control series Zajdel et al. used comprised patients with benign brain neoplasms and diverse neurological disorders, while Baraniskin et al.’s series was dominated by multiple sclerosis cases. Further evaluation of the combined three miRNAs with the same reference groups is required to confirm these findings. Zajdel et al. (17) also revealed that combined miR-let-7b and miR-155 analyses resulted in increased diagnostic accuracy with 96.0% sensitivity and 91.8% specificity. In a word, combined miRNAs panels may improve the sensitivity and specificity of the diagnosis compared with an individual one because one miRNA can target multiple genes and one gene can be regulated by different miRNAs (29).

According to the QUADAS-2 criteria, all the included records displayed moderate to relatively high quality. In addition, we found no evidence of publication bias. These results strengthened the reliability of our findings.

### Limitations

There are some limitations in this study. First, in view of the rarity of PCNSL, the sample size included was small, which may influence the strength of our analysis to some extent. Further validation based on a larger sample of patients and controls is required. Second, the inconsistent identification of an equal cutoff level for miRNAs in all the studies may have an influence on the final results. Third, we did not further conduct meta-regression analysis to explore whether differences in sample size, miRNAs profiled, and specimen type were the potential sources of the interstudy heterogeneity because the number of included studies was insufficient.

## Conclusions

This meta-analysis reveals that miRNAs are suitable as noninvasive diagnostic biomarkers for PCNSL with high accuracy. In addition, both CSF-based and blood-based miRNAs assays for PCNSL detection are considered reliable for clinical application. The miR-21 assays also seem to be more sensitive in the diagnosis of PCNSL. Large sample and good quality studies should be conducted to verify our results. Further studies are required to determine the diagnostic value of blood and CSF miR-21 for PCNSL.

## Data Availability Statement

The original contributions presented in the study are included in the article/**Supplementary Material**, further inquiries can be directed to the corresponding author/s.

## Author Contributions

FC and WL conceived the topic of the study and critically revised it for important intellectual content. XZ searched the literature, analyzed the data, created the figures, and drafted the manuscript. PL and QD searched the literature, analyzed the data, and checked the data extraction. PL and YD undertook the task of drafting and proofreading the manuscript. SY and ZC performed the data extraction. All authors contributed to the article and approved the submitted version.

## Funding

This work was supported by grants from Beijing Municipal Science & Technology Commission (No. Z181100001718127) and Beijing Municipal Health Commission of China (No.2020-2-2048).

## Conflict of Interest

The authors declare that the research was conducted in the absence of any commercial or financial relationships that could be construed as a potential conflict of interest.

## Publisher’s Note

All claims expressed in this article are solely those of the authors and do not necessarily represent those of their affiliated organizations, or those of the publisher, the editors and the reviewers. Any product that may be evaluated in this article, or claim that may be made by its manufacturer, is not guaranteed or endorsed by the publisher.
