# Supplementary figures and images for: MicroRNAs as Diagnostic Biomarkers in Primary Central Nervous System Lymphoma: A Systematic Review and Meta-Analysis
Source: Front Oncol. 2021 Sep 17;11:743542. doi: 10.3389/fonc.2021.743542 (PMC8484918; doi:10.3389/fonc.2021.743542)

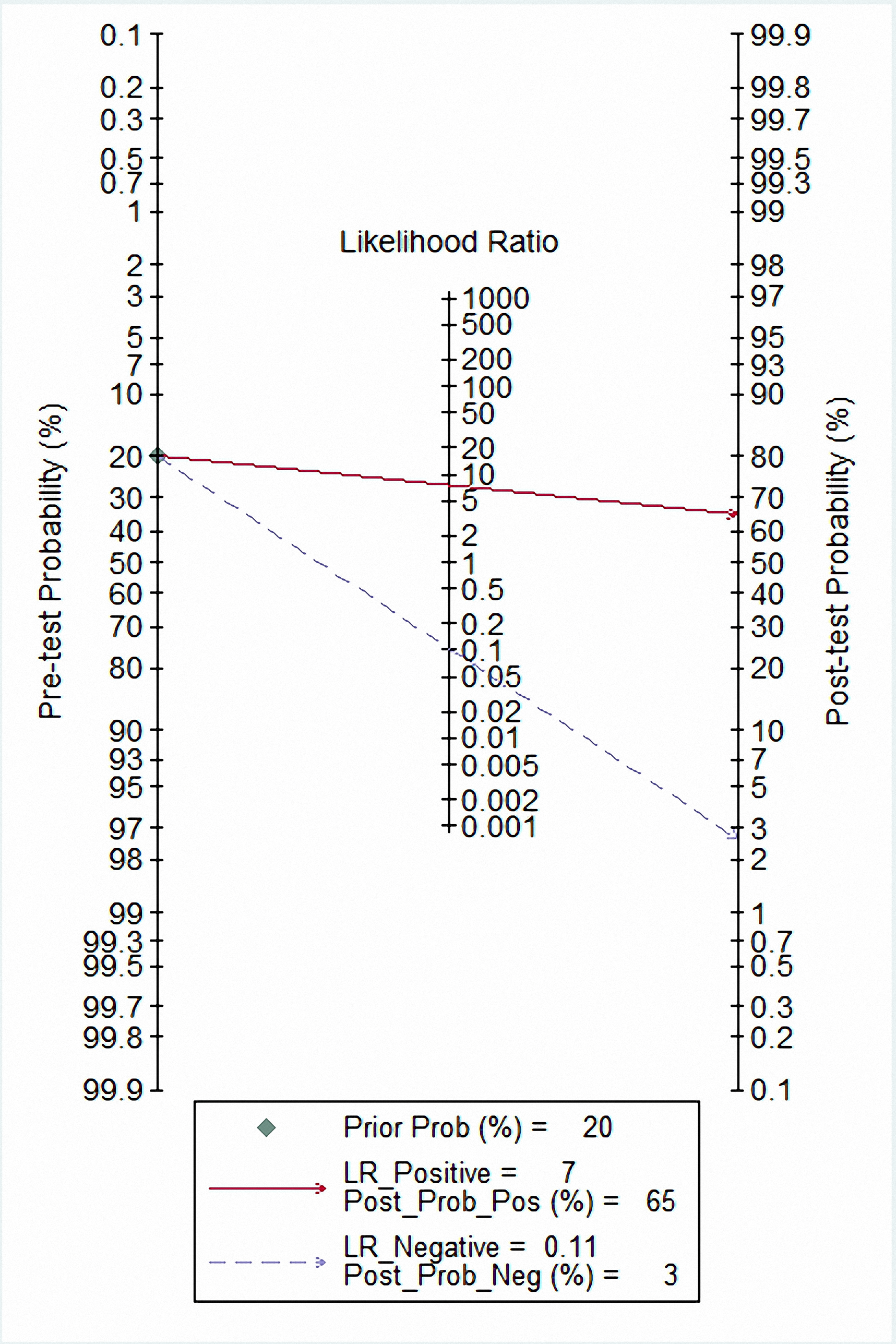

Supplement: Supplementary file 1 [file Image_1.tif]
